# Reactions to Environmental Changes: Place Attachment Predicts Interest in Earth Observation Data

**DOI:** 10.3389/fpsyg.2020.01442

**Published:** 2020-07-10

**Authors:** Marlis Charlotte Wullenkord, Lea Marie Heidbreder, Gerhard Reese

**Affiliations:** Department of Psychology, Social, Environmental, and Economic Psychology, University of Koblenz-Landau, Landau, Germany

**Keywords:** place attachment, Earth observation data, policy support, emotions, pro-environmental intentions, environmental change

## Abstract

Environmental changes such as extreme weather events become increasingly noticeable worldwide. Earth observation (EO) data provide information about such changes, but little is known about citizens’ perceptions of and responses to such changes. Across three studies, we assess whether people’s place attachment on different regional levels predicts interest in EO data, and whether perceived environmental change affects emotional responses and place attachment. Two survey studies (*N* = 118 students and *N* = 197 citizens from the Palatinate in Southern Germany) revealed that place attachment predicts interest in EO data, especially when people felt strongly attached to the region or place in question. A third experimental study (*N* = 600) with repeated measures (*N* = 203) revealed that visualized environmental change – using satellite images of local vs. non-local environmental change – did not affect place attachment but elicited stronger emotional responses than visualizing no change. Policy support measures across Studies 2 and 3 suggest that place attachment and emotional responses are important predictors for action to mitigate consequences of environmental change.

## Introduction

In times of global environmental challenges affecting the qualities of many world regions (IPCC, 2018), it is critical to understand social responses to these largely anthropogenic, biophysical changes. Some of these changes can be observed directly (e.g., flooding, heat-destroyed harvests) but others cannot (e.g., amount of pollutants in the atmosphere), requiring the use of technical instruments and expertise to make these changes visible. Results of these analyses, in turn, need to be processed in a way that people can respond to. In fact, some scholars have argued that action against climate change is not taken because of its often indirect effects and its abstract consequences (Fleury-Bahi, 2008).

It is therefore vital to assess if and how people seek information about environmental change, and what may predict such behavior. We believe that place attachment – the emotional relationship people have to places – may be a strong predictor of interest in environmental change. In this work, we investigate whether place attachment on different levels (regional, national, global) predicts responses to environmental change, and whether such change affects the way people relate to place.

While many regional and global environmental changes are visible and its consequences can be observed directly, a large part of biological, geographical, chemical, and physical processes that define the Earth’s condition remain invisible. The latter can be made visible by data that are acquired from remote sensing platforms such as aircraft, satellites, and surface and subsurface instruments. These so-called Earth observation (EO) data are collected by various private and federal institutions (e.g., European Space Agency), and usually consist of numerical measurements and photos (Bartholome and Belward, 2005; Tomás and Li, 2017). They are used to observe environmental changes, manage and inventory natural resources, and inform the public about the state of the planet. In this research, we test whether people’s place attachment predicts their willingness to engage with EO data. This is important for two reasons: First, because place attachment is related to pro-environmental action (Vaske and Kobrin, 2001; Halpenny, 2010; Cheng and Wu, 2015), and second, because pro-environmental policy support requires at least some understanding of environmental processes.

Place attachment refers to the relationship people have to places. It can be understood as an emotional bond, a sense of belonging of a person to a particular environment that influences how they care for and attend to that environment (Lewicka, 2011b; Devine-Wright, 2013). Korpela (2012) describes it as a mutual relationship between a person and its environment, making a space a place people identify with.

Place attachment is a multi-faceted construct that can be defined at different levels of abstraction. One may (simultaneously) relate to one’s local place of residence (e.g., neighborhood, city; local place attachment), to a larger region (e.g., region, country; regional place attachment), and even to the whole planet (global place attachment; Devine-Wright, 2013; Walker et al., 2015). With increased mobility and education, people report changes in place attachment with reference to the scale of place that they attach to, such as from the local to the European level (Tuan, 1977; Gustafson, 2009). At the same time, previous studies suggest that global place attachment in particular seems to predict support for adaptation policies (Walker et al., 2015). Yet unexplored, however, is whether an interest in EO data or mitigation policies referring to different levels (such as policies regarding local, national, or global changes) depend on the levels of place attachment.

People can also relate to places in qualitatively different ways. Based on Hummon (1992), Lewicka (2011a) distinguishes between traditional and active place attachment. Traditional place attachment refers to an established way of living, in which people are attached because they have lived in a place for long, are not very mobile, and have little comparison with other places. This understanding of place attachment mirrors literature suggesting that strong social ties and duration of residency are the strongest predictors of place attachment (e.g., Kasarda and Janowitz, 1974; Lewicka, 2011b). Active place attachment, however, reflects a more conscious way of relating with place, characterized by an interest in what happens in the place and how the place develops and changes (Lewicka, 2011a). Another conceptualization differentiates place identity from place dependence (Williams and Vaske, 2003). Place identity refers more strongly to the cognitive-emotional bond to a place (i.e., the way in which one relates a place to one’s self and identity). Place dependence reflects the behavioral bond to the place (i.e., what one can or cannot do in the place, compared to other places).

These different facets notwithstanding, there is a core to place attachment as such – the strong bond to a place. In the current set of studies, we seek to do justice to these different conceptualizations and the inherent core of place attachment, using both multidimensional measures as well a short, visual measure that provides information about a person’s place attachment in a parsimonious manner.

Current research demonstrates partially contradicting results concerning the relation between place attachment and pro-environmental behavior (Scannell and Gifford, 2010). Pro-environmental behavior can be understood as any environmentally protective behavior that individuals exhibit in relation to their natural environment, behavior that causes as little damage to the environment as possible (Steg et al., 2013). In many cases place attachment is strongly connected to pro-environmental behavior (Vaske and Kobrin, 2001; Cheng and Wu, 2015; Devine-Wright et al., 2015; Walker et al., 2015), but evidence is still mixed (for an overview, see Scannell and Gifford, 2010), both with regard to physical place attachment (referring to physical characteristics of a place such as buildings or natural environment) and to social place attachment (referring to interpersonal relationships such as neighborhood attachment). Other studies (e.g., Uzzell et al., 2002) show a weak negative relation between place identity and frequency of pro-environmental behavior.

Concerning awareness of environmental problems, people with high place identity rated problems such as crowding, litter, or noise more negatively whereas people with high place dependence rated these problems as less important (Kyle et al., 2004). Similarly, in a group of youth working in a natural resource program, place identity was related to pro-environmental behavior, and mediated the relation of place dependence and pro-environmental behavior (Vaske and Kobrin, 2001). Research on risk perception – an intuitive judgment of risk (Slovic, 1987) – suggests that place attachment also relates to risk perceptions and coping behavior (for a review, see Bonaiuto et al., 2016). For example, place attachment moderated the relation between risk perception and coping with an environmental risk, such that for people with high place attachment, risk coping intentions decreased (De Dominicis et al., 2015).

Many studies explored the role of place attachment in the context of *NIMBYism* (Devine-Wright, 2009) as place attachment is related to participation in community projects (Manzo and Perkins, 2006). Those residents who expressed high place attachment to *specific* areas of their community were more opposed to the development of hydropower plants whereas those with high *overall* place attachment supported the development (Vorkinn and Riese, 2001). Vorkinn and Riese (2001) also found a negative relation between place attachment and evaluation of an energy project. Apart from regional bonds, global place attachment and identity had a strong connections to pro-environmental behavior, climate change opinions, and policy support (Devine-Wright et al., 2015; Walker et al., 2015).

Given that place attachment is an emotional bond to a place, it is likely that emotional experience may also be related to environmental changes. While there is, to our knowledge, only little research addressing emotional responses to environmental change (e.g., Doherty and Clayton, 2011; for an overview, see Leiserowitz, 2006), research findings suggest that both positive (e.g., hope; Ojala, 2012) and negative (e.g., guilt, worry; Harth et al., 2013; Smith and Leiserowitz, 2014) emotions may predict pro-environmental behavior. For example, Reese and Jacob (2015) analyzed a large representative survey and found that experiencing anger – usually termed a negative emotion – was positively related to pro-environmental action (see also Harth et al., 2013; Rees et al., 2015; for guilt). Ojala (2012) found that hope – usually termed a positive emotion – predicted pro-environmental action. Importantly, emotions can mediate the relation between certain perceptions, such as risk, and actual behavioral action (e.g., Böhm and Pfister, 2000). We address emotional responses to environmental change in Study 3.

Taken together, the goal of the current research is to identify whether the use of EO data can inform social science research on perceptions and experience of place. There is some experimental research suggesting that (perceived) changes in place may alter place attachment (Scannell and Gifford, 2017; Reese et al., 2019). Thus, place attachment may represent an important psychological concept that links changes in the biophysical environment to actual behavioral responses of citizens. Investigating how people perceive, appraise and act in their local and global communities, vis-á-vis environmental change, we assess place attachment as a function of the aforementioned technologies. The primary research questions are thus (1) whether place attachment on various levels (e.g., local, regional, global) relates to greater interest in EO data, (2) whether specific EO data about a specific region alters individuals’ perceptions of place and emotions, and (3) whether visualized EO data affect citizens’ efforts to change behavior.

We address these questions in a pilot study and two fully developed studies. The pilot study presents a first approach as to whether there is a relation between place attachment and interest in EO data. In Study 2, we use a correlational approach to test whether place attachment on various levels relates to greater interest in and use of EO data on various levels. In Study 3, we use a repeated-measures experiment to test whether specific EO information may affect people’s place attachment, emotional responses to environmental change, climate policy support, and pro-environmental intentions.

## Study 1: Pilot Study

We conducted a brief pilot study in order to explore whether place attachment may be related to interest in EO data addressing environmental change and intention to search for relevant information. One-hundred-eighteen students from the Palatinate in the South of Germany signed informed consent and participated in this study. It was conducted during the first session of a psychology lecture and handed out on a one-page questionnaire. Participants were *M* = 21.23 years old (*SD* = 2.65), and 87 self-identified as female, 31 as male.

Place attachment was measured with a 12-item scale based on work by Williams and Vaske (2003), including six items measuring the sub-dimension place identity (e.g., “I feel the Palatinate is a part of me,” α = 0.94) and six items measuring place dependence (e.g., “The Palatinate is the best place for what I like to do,” α = 0.76). Participants responded on 7-point Likert scales from “1 – strongly disagree” to “7 – strongly agree.” For overall place attachment, the mean of both subscales was computed (α = 0.91). We then asked participants to indicate their interest in data addressing environmental change (“I am interested in how the Palatinate will change due to atmospheric changes”) and information search (“I would search information that explains the impact of pollutants in the Palatinate”). Finally, socio-demographic variables were collected (i.e., gender, age, length of residence in the place).

Place attachment – both the complete scale and the subscales – correlated significantly with both interest and information search (Table 1). The stronger place attachment, the more participants expressed interest in data that explain environmental change, such as EO data do, and the more willingness they expressed to search related information.

**TABLE 1:** Correlations of place attachment scales, interest, and information search (*N* = 118).

	Scale	1	2	3	4	5
1	Overall PA	1				
2	Place identity	0.94***	1			
3	Place dependence	0.86***	0.65***	1		
4	Interest in EO data	0.45***	0.48***	0.31*	1	
5	Information search	0.35***	0.34***	0.27*	0.64***	1

*p < 0.05; ***p < 0.001. EO = Earth Observation.

Further inspection of the data, using multiple regression analysis with place identity and place dependence as independent predictors (controlling for age, gender, and length of residence), suggests that place identity is a more important predictor of interest and information search than place dependence (see Tables 2A,B).

**TABLE 2A:** Regression analysis for interest in Earth Observation data (*N* = 118).

Variables	*B*	β	*SE*	*p*	*R*^2^	Adjusted *R*^2^
(Constant)	2.882		1.339	0.034*	0.25	0.21
Place identity	0.615	0.553	0.141	< 0.001***		
Place dependence	–0.035	–0.021	0.187	0.851		
Duration of residence	–0.002	–0.096	0.002	0.350		
Age	–0.051	–0.076	0.055	0.360		
Gender	0.276	0.069	0.329	0.403		

*p < 0.05; ***p < 0.001.

**TABLE 2B:** Regression analysis for intention to search information (*N* = 118).

Variables	*B*	β	*SE*	*p*	*R*^2^	Adjusted *R*^2^
(Constant)	2.991		1.366	0.031*	0.14	0.10
Place identity	0.361	0.341	0.143	0.013*		
Place dependence	0.113	0.069	0.190	0.553		
Duration of residence	–0.001	–0.067	0.002	0.545		
Age	–0.049	–0.077	0.056	0.385		
Gender	0.261	–0.069	0.336	0.438		

*p < 0.05.

Taken together, first cautious findings suggest that place attachment may be an important predictor of how people – more precisely, in Study 1, students – respond to changes in the environment and interest in data that explain such changes. The latter could be communicated by EO data (e.g., showing landscape alteration caused by climate change). Building on this preliminary finding, we conducted two studies to further explore this relation, focusing on different levels of place abstraction.

## Study 2: Correlational Study

### Aim and Hypotheses

Study 2 aimed at investigating whether place attachment at various levels (regional, national, global) relates to greater interest in EO data. We expected people with high regional place attachment to be more interested in EO data of places in their region. In line with previous research (Walker et al., 2015), people with high national place attachment should be more interested in EO data of their country than in global changes. People who feel attached to the whole planet should be interested in global EO data. Furthermore, we examined the relation between place attachment and policy support. Previous research suggests that stronger place attachment relates to stronger preference for place-protecting policies (Walker et al., 2015). Consequently, stronger place attachment should also relate to interest in environmental changes that require such policies.

### Method

#### Sample

Study 2 was realized in summer 2018 on the Platform SoSci-Survey (Leiner, 2016). Participants were recruited via street surveys in different (rural and urban) areas of the Palatinate in the South of Germany. They were approached by a female experimenter who handed a tablet computer to participants for recording the data. For compensation participants could win a shopping voucher of a local store. One-hundred ninety-seven participants from the Palatinate completed the survey. This region is characterized by tree-covered hill landscapes with scattered settlements throughout the forest but also intensive land-use (wine production) between the forest limits and the large Rhine basin. Mean age was *M* = 41.86 years (*SD* = 13.66) with an age range from 19 to 79; 57% were female and 40% male. Education level was above the national average (Destatis, 2018): 51% reported to have a university degree (18% on the national level) and 28% to hold a high school diploma (32% on the national level). On average, participants had lived in the region for *M* = 32.62 years (*SD* = 18.65) with a range from 1 to 79 years.

#### Materials

The survey included measures of place attachment, interest in EO data, policy support, environmental consciousness, and political orientation. If not otherwise stated, participants answered all items on a 7-point-Likert-scale from “1 – strongly disagree” to “7 – strongly agree.” All participants gave their written informed consent to participate at the beginning of the study.

##### Place attachment

We measured place attachment on three different levels (regional, national, global). Each scale corresponding to a level of analysis comprised six items of place identity (Williams and Vaske, 2003; e.g., “I identify strongly with the Palatinate”). We excluded the dimension of place dependence as place identity appeared to be the stronger predictor of interest in EO in Study 1. To take the multifaceted nature of place attachment into account (Scannell and Gifford, 2010), we added physical and social connectedness (Scannell and Gifford, 2010; Reese et al., 2019) as further dimensions. They were measured with eight items for the regional and national level and six items for the global level [e.g., “The natural environment in the (place) means a lot to me” or “I feel connected with the people in the (place)”]. Exploratory factor analysis revealed no difference between place identity and physical or social connectedness, thus we collapsed them into one scale for regional place attachment (α = 0.96), national place attachment (α = 0.96), and global place attachment (α = 0.95).

##### Interest in EO data

Participants read a short introduction about EO data and were then asked about their interest in “information about climatic and scenic changes in (place)” with six items for each level (regional, national, global; e.g., “I would be interested in such information” or “I would tell my friend about such information”). Items were generated based on discussions with environmental scientists and psychologists. Internal consistency was excellent: α = 0.91 (regional), α = 0.90 (national), α = 0.90 (global).

##### Policy support

To measure policy support we asked participants how strongly they would support different political measures. Eight items were formulated. For example, “Tax on consumer goods that have an excessively high carbon footprint” or “Investment in global climate protection measures” (α = 0.93). These items were generated and adapted from Loy and Reese (2019).

##### Socio-demographics

Apart from age, gender, education level, and duration of residence we measured *Environmental Consciousness* and *Political Orientation* as control variables. We expected political orientation to relate to policy support (Dietz et al., 2007; Ziegler, 2017) and thus included it as a control variable. To measure environmental consciousness participants responded to the item “I am an environmentally conscious person” on a slider bar ranging from “1 – strongly disagree” to “5 – strongly agree.” Participants indicated their political orientation on a slider bar ranging from “1 – left” to “101 – right,” following previous standard procedures (e.g., Drews and Reese, 2018). The instruction read as follows “In politics, people sometimes talk about ‘left’ and ‘right.’ Where would you place yourself on the following scale?”

We used Microsoft Powerpoint to create all figures and graphics presented in this article.

### Results

#### Descriptives

Correlations, means, and standard deviations of the variables are displayed in Table 3. *t*-tests revealed regional place attachment to be stronger than national place attachment (*t*[392] = 3.70, *p* < 0.001) but equal to global place attachment (*t*[392] = −1.41, *p* = 0.158), and global place attachment was stronger than national place attachment (*t*[392] = 5.20, *p* < 0.001). Participants tended to be more interested in regional than in global EO data (*t*[392] = 1.88, *p* = 0.061). Furthermore, people with strong global place attachment showed higher policy support. Regional and national place attachment were unrelated to policy support.

**TABLE 3:** Correlations, means, and standard deviations of all place attachment scales.

	PA	*M*	*SD*	1	2	3	4	5	6	7	8	9
1	PA regional	5.25	1.36	1								
2	PA national	4.76	1.30	0.61***	1							
3	PA global	5.45	1.32	0.27***	0.44***	1						
4	IEO regional	5.10	1.32	0.46***	0.42***	0.48***	1					
5	IEO national	4.90	1.36	0.26***	0.37***	0.50***	0.89***	1				
6	IEO global	4.85	1.31	0.25***	0.33***	0.55***	0.85***	0.93***	1			
7	PS	5.73	1.23	0.05	0.10	0.40***	0.36***	0.37***	0.37***	1		
8	PO	39.83	20.89	0.19*	0.25***	−0.13*	–0.05	–0.07	–0.11	−0.29***	1	
9	EC	3.98	0.75	0.21***	0.15*	0.35***	0.38***	0.41***	0.45***	0.31***	–0.08	1

*p < 0.05; ***p < 0.001. (*N* = 197), PA, place attachment; IEO, Interest in Earth Observation Data; PS, Policy Support; PO, Political Orientation; EC, Environmental Consciousness.

#### Results of Regression Analysis: Predicting Interest in EO Data

**FIGURE 1:**
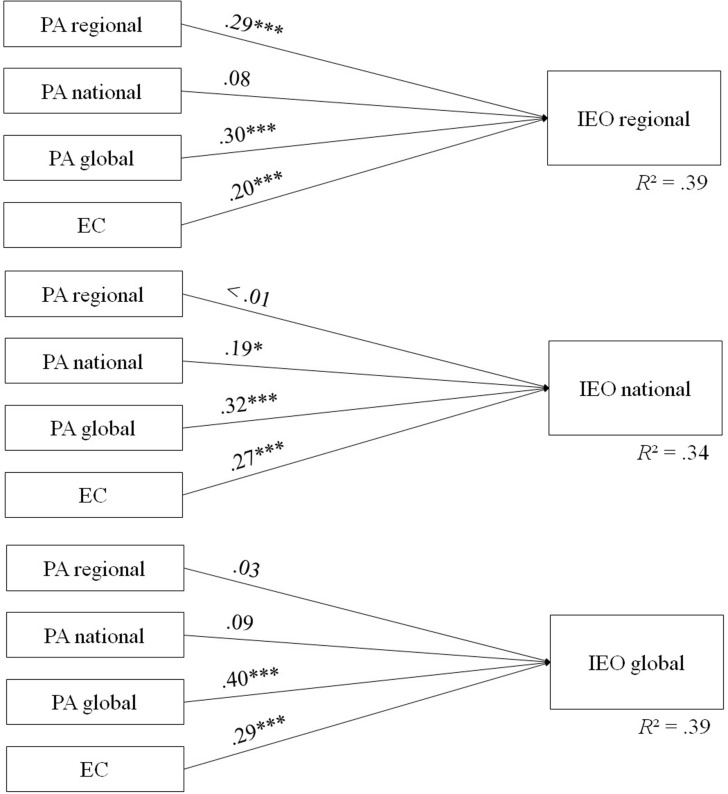
Model of antecedents for the three levels of IEO (*N* = 197). IEO, Interest in Earth Observation Data; PA, Place attachment; EC, Environmental Consciousness. *p < 0.05, ***p < 0.001.

**FIGURE 2:**
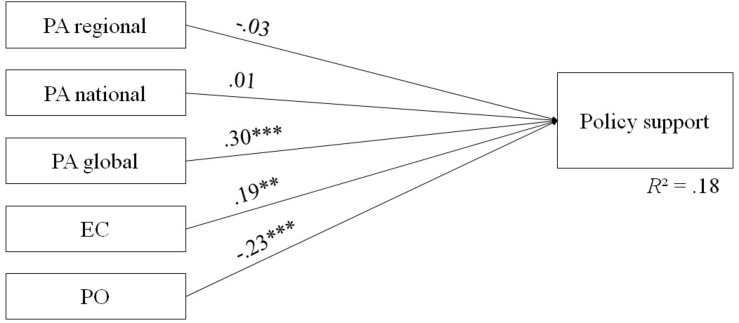
Model of antecedents for policy support (*N* = 197). PS, Policy Support; PA, Place attachment; EC, Environmental Consciousness; PO, Political orientation. **p < 0.01, ***p < 0.001.

Hierarchical stepwise regression was performed to predict interest in EO data. In a first step, socio-demographics (gender, age, duration of residence) and control variables (political orientation, environmental consciousness) were included. Socio-demographics and political orientation were no significant predictors for interest in EO data on all three levels. Thus, only environmental consciousness and place attachment were included in the analysis (Figure 1). Global place attachment was the strongest predictor for interest in EO data on each level. However, regional place attachment had additional impact on interest in regional EO data and national place attachment for national EO data. Place attachment was an important predictor for interest in EO data beyond environmental consciousness. Both political orientation and environmental consciousness were significant predictors of policy support and were thus included in the final analysis. Results revealed global place attachment as the strongest predictor beyond policy orientation and environmental consciousness. Regional and national place attachment were not related to policy support (Figure 2).

### Discussion

Study 2 suggests that place attachment is related to stronger interest in EO data. Even though we cannot infer causality, relations between high regional place attachment and stronger interest in regional EO data may suggest that a bond to one’s own region might lead to interest in change and condition of one’s own region. The same patterns were found for the national and global level. However, exceeding regional and national boundaries, global place attachment predicted interest for EO data on all levels. Global place attachment showed a stronger relation to environmental consciousness than regional or national place attachment. The strong relation between global place attachment and interest in EO data should be interpreted against these findings. The climate crisis and its negative impacts might be mostly seen as a global issue. Remarkably, place attachment could explain variance in interest in EO data beyond environmental consciousness. Regional and national place attachment did not relate with policy support although it was operationalized on different geographical levels. This is in line with previous literature finding global identity – a concept closely related to global place attachment – to be an important predictor for pro-environmental behavior and policy support (Rosenmann et al., 2016; Joanes, 2019; Loy and Reese, 2019; for an overview, see McFarland et al., 2019).

Both Study 1 and Study 2 suggest that place attachment and interest in EO data are related. In the third study, we sought experimental evidence, testing whether objective changes in EO data-based visualizations result in changes in place attachment and emotional reactions.

## Study 3: Experimental Study

### Aim and Hypotheses

The third study employed an experimental repeated-measures design to test whether information derived from EO data affects feelings of place attachment, emotions, policy support, and pro-environmental intentions. We tested whether observing one’s place or a remote place deteriorating as a result of climate change would alter the extent of place attachment people report. We conducted the study in the same region as Study 2, however, in different communities to avoid sample dependence. Deterioration was visibly very apparent in the Palatinate for the first time in the summer of 2018 when the study was conducted. Previous research suggests that merely imagining changes of place can alter perceptions of place (Reese et al., 2019). Similarly, in the current experiment we expected that place attachment would decrease when people were confronted with negative EO information (i.e., fruitful land that became arid) – however, only with regard to their actual place of residence. Further, we expected people in the change conditions to react with stronger negative emotions toward the change. In particular, participants in the condition depicting change of their home region should report more negative emotions compared to those seeing no change, or change in another place. Finally, we expected a change in policy support and environmental intentions as a function of experimental condition. People in the change conditions should support environmental policies more than people in the no change conditions, and report stronger pro-environmental intentions.

### Method

#### Sample

Six hundred participants from the Palatinate (*M*_*age*_ = 35.14 years; *SD_*age*_* = 16.30; *n* = 405 identified as female, *n* = 187 as male), completed an online survey. Participants who indicated to live in other areas than the Palatinate were excluded from the study. On average, duration of residence in the region was *M* = 24.96 years (*SD* = 19.11), ranging from zero to 80 years. The sample had higher education than the general population in the area: 34% had a high school diploma and 36% a university degree. Of those, 211 participated in a second part of the study 2 weeks later (*M*_*age*_ = 34.94 years; *SD_*age*_* = 14.66; *n* = 140 identified as female, *n* = 65 as male, one chose not to indicate gender).

#### Procedure

**FIGURE 3:**
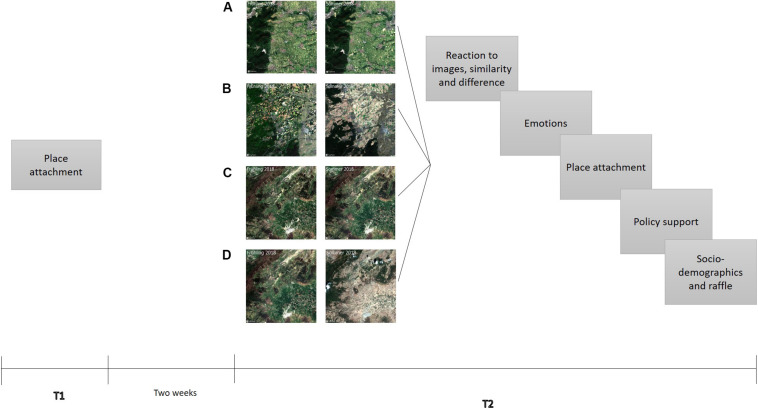
Graphic scheme of the experimental procedure including satellite images depicting **(A)** no change and **(B)** a change in vegetation in participants’ local environment and a control region in Tunisia depicting **(C)** no change and **(D)** a change in vegetation. Images were labeled as “Frühling” (spring) and “Sommer” (summer) 2018. Images were recorded from the application “Snap Planet” by the European Space Agency.

Figure 3 depicts a graphic scheme of the experimental procedure. The survey was implemented online using SoSci-Survey (Leiner, 2016). Participants were mainly recruited face-to-face using tablets in various locations in the Palatinate (68%) or via social media (25%). All participants gave their written informed consent at the beginning of the study. We used the first assessment point to measure a baseline level of place attachment, using different measures of place attachment. Around 2 weeks later, participants could choose to participate in the second part of the study. In the second part, EO data were presented to people in the form of satellite images depicting no change or change of either their home region or a control region in a country with which most participants were assumed not to have a strong relation. We showed people in two conditions images of a control region that we expected to influence people’s global place attachment, rather than their local place attachment. Specifically, we randomly assigned participants to one of four experimental conditions: (a) local region (= home region) without change, (b) local region with change, (c) global region (= control region) without change, (d) global region with change. The European Space Agency routinely takes images of the Earth and makes them accessible to the public through their App “Snap Planet,”^1^ which we used to record the images. We told participants that we were showing them images from spring and summer of 2018 (the year of data collection) and asked them to observe the images carefully. The images either depicted a drastic change between the seasons (green landscape vs. drought, Figures 3B,D), which was realistic, or no change (green landscape vs. manipulated green landscape, Figures 3A,C), which was manipulated. The images contained similar amounts of green and brown between the regions, respectively. Such a drastic change in vegetation between the seasons is normal in the control region. However, in the Palatinate where the study took place it is interpreted as an extreme weather event, showcasing climate change in people’s “backyards” and suggesting that their place could become less habitable over time (Reinermann et al., 2019). After familiarizing themselves with the images, participants wrote about their spontaneous reactions to seeing the images in an open-ended question^2^ and indicated whether they perceived the two images to be similar or different. They then answered questions about emotions, place attachment to the Palatinate and the Earth as a whole, support for different climate relevant policies implemented both at the regional and global level, and socio-demographic and control variables.

As a compensation for participating in both parts, participants could take part in a raffle for twenty cinema vouchers (worth 20€ each). In the very end, they were debriefed about the deception and the real purpose of the study. The local ethics committee granted ethical approval for the study (153_2018).

#### Materials

The survey at T1 measured different qualities and scales of place attachment at the local and global level, socio-demographic (age, gender, duration of residence) and control (environmental consciousness and political orientation) variables. National place attachment was no longer assessed as it did not prove predictive of outcome variables in Study 2. At T2, we also measured emotions and policy support. Again, if not otherwise stated, participants answered all items on a 7-point-likert-scale from “1 – strongly disagree” to “7 – strongly agree.”

##### Visual single item measure of place attachment (SIMPLA)

A new visual scale of place attachment was implemented and tested in this study. The scale consists of a slider bar on which a stick figure can be moved to indicate the relationship one has with a place. Place was symbolized on the right, using either an image of planet Earth or the Palatinate. Placing the stick figure close to or inside the image was interpreted as indicating a close relationship.

##### General place attachment

General place attachment was measured at the regional and global scale (emotional bond to the region and to planet Earth, respectively). In both cases, four items from the *Place Attachment Scale* (Williams and Vaske, 2003) were used [e.g., “I identify strongly with the Palatinate,” α_*reg*_ = 0.94, α_*glo*_ = 0.92; α_*Williams*_ = (0.84–0.94)].

##### Active and traditional place attachment

In order to lend more generalizability and reliability to our research question we decided to use an alternative measure of place attachment in Study 3. Specifically, we now focused on active and traditional place attachment to the Palatinate. It was measured with the subscales *Place Inherited* (corresponding to traditional place attachment) and *Place Discovered* (corresponding to active place attachment) of the *Relations with the City/Town/Village scale* (Lewicka, 2011a, b; based on Hummon, 1992). Traditional place attachment was measured using three items (e.g., “I can’t imagine leaving the Palatinate for good,” α_*trad*_ = 0.75, α_*Lewicka*_ = 0.76). Active place attachment was measured using four items (e.g., “I like to visit and discover new places in the Palatinate,” α_*act*_ = 0.84, α_*Lewicka*_ = 0.72).

##### Emotions

Based on the PANAS (Watson et al., 1988; German translation: Krohne et al., 1996) and on previous literature (Fritsche et al., 2010; Jugert et al., 2016), six emotions (concern, anger, guilt, helplessness, joy, hope) felt after looking at the satellite images were judged as most relevant in the context of environmental change. The instructions read: “After looking at the images, I feel…”

##### Policy support and pro-environmental intentions

We measured support for different climate relevant policies implemented both at the regional and global level with four items asking participants how much they would support different policies (e.g., “increasing taxes for local business that emit a lot of CO_2_”). The measure had acceptable internal consistency (α = 0.79).

We also used four items to ask participants in how far they intended to engage in politics in the future to limit climate change (e.g., “I plan to become involved with politics in the future to limit the consequences of climate change in the Palatinate”). We also asked whether they intended to change their everyday lives to tackle climate change (e.g., “I plan to act in an environmentally protective way in my everyday life in the future to limit the consequences of climate change in the Palatinate”). Internal consistency of the measure was good, α = 0.83.

#### Data Preparation and Statistical Analysis

Four of the cases in T2 could not be matched with cases in T1 and were deleted. Using Mahalanobis distance, we identified no cases as multivariate outliers at T1 and 4 at T2 and excluded them from further data analyses, leaving a final sample of *n* = 600 at T1 and *n* = 203 at T2.

### Results

#### Descriptives

Means, standard deviations, and correlations of all scales at T1 and T2 are displayed in Tables 4A,B. At T1, global place attachment was higher than regional place attachment, both when measured using general place attachment (*M*_*reg*_ = 5.12, *SD*_*reg*_ = 1.60; *M*_*glo*_ = 5.72, *SD*_*glo*_ = 1.16) and SIMPLA (*M*_*reg*_ = 67.11, *SD*_*reg*_ = 26.47; *M*_*glo*_ = 71.16, *SD*_*glo*_ = 20.74). Distributions were skewed to the left for all measures of place attachment, *sk*_*reg*_ = (−1.05 – [−0.66]), revealing that the majority of participants reported high regional and global place attachment. These patterns were replicated at T2 (Table 4A). People who reported longer residency indicated higher regional place attachment both on the general place attachment scale (*r* = 0.56) and SIMPLA (*r* = 0.41). Correlation between duration of residence was stronger with traditional place attachment (*r* = 0.51) than with active place attachment (*r* = 0.39). Place attachment and political orientation correlated weakly. At T1, people who indicated being on the left of the political spectrum reported higher levels of global place attachment (*r*_*GPA–T*__1_ = −0.12), whereas people who indicated being on the right of the political spectrum reported higher levels of attachment to the Palatinate (*r*_*GPA–T*__1_ = 0.14, *r*_*SIMPLA–T*__1_ = 0.15, *r*_*traditional–T*__1_ = 0.18). Across time and dimensions, the more place attachment people reported, the more they also indicated to be environmentally conscious (*r* = [0.09–0.41], Table 4A).

**TABLE 4A:** Correlations, means, and standard deviations of place attachment, policy support, pro-environmental intentions and socio-demographics at T1 and T2.

		*M_*T1*_ (SD_*T1*_)*	*M_*T2*_ (SD_*T2*_)*	1	2	3	4	5	6	7	8	9	10	11
1	GPA regional	5.15 (1.60)	5.24 (1.49)	1	0.66***	0.16	0.04	0.64***	0.69***	0.06	0.03	0.33***	0.11	0.08
2	SIMPLA regional	67.11 (26.47)	69.74 (23.05)	0.63***	1	0.20***	0.44***	0.45***	0.47***	0.09	0.05	0.18	0.03	0.18
3	GPA global	5.72 (1.16)	5.73 (1.09)	0.19***	0.15***	1	0.61***	–0.07	0.24***	0.39***	0.49***	0.23*	–0.20	0.41***
4	SIMPLA global	71.16 (20.47)	69.91 (20.93)	0.09*	0.49***	0.51***	1	–0.08	0.15	0.30***	0.30***	0.09	–0.25	0.34
5	PA regional traditional	4.01 (1.78)	3.94 (1.76)	0.66***	0.43***	0.02	–0.02	1	0.37***	–0.03	–0.03	0.37***	0.12	0.03
6	PA regional active	5.48 (1.25)	5.63 (1.06)	0.72***	0.47***	0.33***	0.17***	0.49***	1	0.12	0.18**	0.31***	0.01	0.18***
7	Policy support	-	4.69 (1.19)	−	−	−	−	−	−	1	0.45***	0.07	−0.29***	0.37***
8	Pro-environmental intentions	-	6.03 (1.02)	−	−	−	−	−	−	−	1	0.12	−0.29***	0.34***
9	Age	35.14 (16.30)	34.67 (14.50)	0.37***	0.22***	0.34***	0.16***	0.35***	0.41***	−	−	1	0.01	0.12
10	political orientation	38.43 (19.82)	36.64 (17.67)	0.14***	0.15***	−0.12***	–0.06	0.18***	0.06	−	−	0.10*	1	−0.19**
11	environmental consciousness	77.04 (18.72)	76.31 (17.16)	0.14***	0.17***	0.38***	0.25***	0.09*	0.24***	−	−	0.30***	–0.03	1

T1 (*N* = 600) below the diagonal, T2 (*N* = 203) above the diagonal. *N*’s may differ slightly due to missing values on some of the socio-demographic variables. GPA, general place attachment; PA, place attachment; SIMPLA, visual place attachment. *p < 0.05; **p < 0.01; ***p < 0.001.

**TABLE 4B:** Correlations, means, and standard deviations of emotions with variables at T2.

		*M (SD)*	1	2	3	4	5	6	7	8	9	10	11	12	13	14	15	16	17
1	Worry	4.35 (2.09)	1						–0.03	0.10	0.16*	0.22***	0.00	0.05	0.20**	0.18**	–0.07	0.01	0.16*
2	Anger	3.06 (1.90)	0.66***	1					–0.08	0.09	0.12	0.19**	0.05	–0.05	0.22***	0.25***	–0.12	−0.14*	0.16*
3	Guilt	2.82 (1.64)	0.59***	0.74***	1				–0.01	0.10	0.12	0.11	0.07	0.06	0.18**	0.21***	–0.10	–0.04	0.13
4	Helpless-ness	3.51 (2.00)	0.64***	0.63***	0.59***	1			0.03	0.09	0.09	0.12	0.12	0.03	0.13	0.16*	–0.08	–0.03	0.08
5	Joy	2.23 (1.63)	−0.33***	–0.05	0.01	–0.05	1		0.13	0.08	0.00	–0.04	0.07	0.04	0.02	–0.02	0.00	–0.11	–0.09
6	Hope	2.55 (1.67)	−0.25***	0.03	0.05	0.02	0.76***	1	0.09	0.08	–0.02	–0.02	0.02	0.05	0.02	0.00	–0.08	–0.09	–0.08

*N* = *203*, 7 = general place attachment regional, 8 = visual place attachment (SIMPLA) regional, 9 = general place attachment global, 10 = SIMPLA global, 11 = place attachment regional traditional, 12 = place attachment regional active, 13 = Policy support, 14 = Pro-environmental intentions, 15 = Age, 16 = political orientation, 17 = environmental consciousness. *p < 0.05; **p < 0.01; ***p < 0.001.

#### Effect of the Intervention: Comparisons Between Conditions

At T1, we observed no difference between the four groups with respect to place attachment, *F*(3,199) = (0.166–0.817), *p* > 0.05. The groups also did not differ with respect to socio-demographic variables, *F*(3,[195–199]) = (0.164–0.955), *p* > 0.05, meaning that groups did not differ before random assignment to experimental conditions at T2. Opposing our hypothesis, there was no observable effect of the intervention on all dimensions of place attachment [place attachment at T2, *F*(3,199) = (0.334–759), *p* > 0.05, and discrepancy scores of place attachment between T1 and T2, *F*(3,199) = (0.663–1.829), *p* > 0.05)] and pro-environmental intentions, *F*(3,202) = 1.271, *p* > 0.05. There was only a marginal effect of the intervention on the discrepancy score of the regional SIMPLA, *F*(3,199) = 2.24, *p* = 0.085. A *post hoc* Tukey-test revealed a trend, indicating that those who did not see their local environment changing (condition a) scored lower on the regional SIMPLA than those who did not see a change in a control region (condition c, *p* = 0.071, *M* = −0.25 vs. *M* = 0.25). An ANOVA revealed an observable trend of an effect of the intervention on reported policy support, *F*(3,202) = 2.62, *p* = 0.052. A *post hoc* Tukey-test revealed a significant difference between the conditions local change (condition b) and no local change (condition a) at *p*_*adj*_ < 0.05, with those observing no local change reporting significantly higher levels of support for climate relevant policies (*M* = 5.06) than those who observed a change on the local level, meaning seeing their local region unusually dry (*M* = 4.46). This finding warrants further discussion.

**FIGURE 4:**
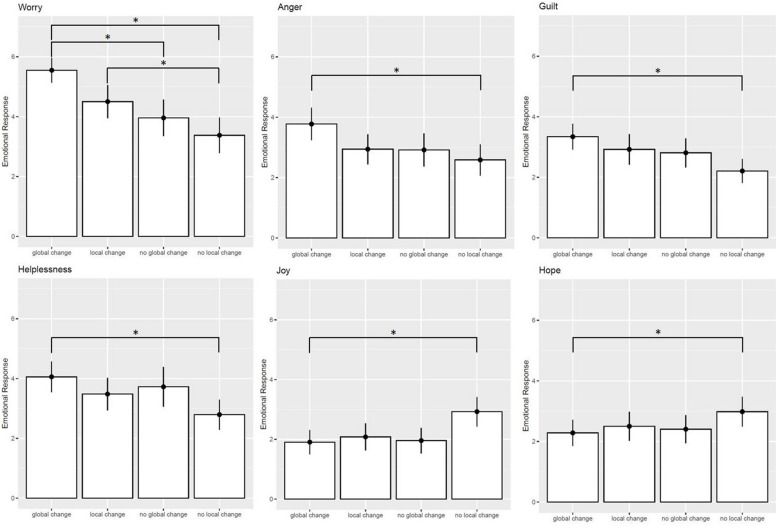
Emotional responses across study conditions.

Importantly, all emotions except for feelings of hope were significantly affected by the intervention (Figure 4). *Post hoc* Tukey-tests (*p*_*adj*_ = 0.05) showed the biggest difference between those who saw a control region changing (condition d) opposed to those who did not observe their local environment changing (condition a). Those who observed a drastic change in the control region reported more feelings of worry (*M* = 5.55 vs. *M* = 3.39), anger (*M* = 3.77 vs. *M* = 2.63), guilt (*M* = 3.34 vs. *M* = 2.22), and helplessness (*M* = 4.06 vs. *M* = 2.81), and less feelings of joy (*M* = 1.91 vs. *M* = 2.91) when looking at the satellite images, compared to those observing no change in their home region (condition b). People observing a drastic change in their home region also reported more feelings of worry (*M* = 4.49 vs. *M* = 3.39) and there was a trend indicating that they reported less feelings of joy (*p*_*adj*_ = 0.054, *M* = 2.12 vs. *M* = 2.91) when compared to the group that observed no change their local environment (condition a). When people looked at satellite images showing a change in the control region (condition d), they also reported more feelings of worry (*M* = 5.55) when compared with those who did not observe a change in the control region (condition c, *M* = 4.04) or those who observed a change in their local environment (condition b, *M* = 4.49).

#### Place Attachment, Policy Support, and Pro-environmental Intentions

To examine the relations between initial place attachment and policy support and pro-environmental intentions, respectively, we calculated multiple regression analyses. Both policy support and pro-environmental intentions could be predicted using the global place identity subscale and political orientation. Environmental consciousness also predicted policy support. Tables 5, 6 depict the results of the analyses, indicating that the more people identify with planet Earth and the more politically left they report being, the more they are willing to support pro-environmental policies and the more pro-environmental intentions they report.

**TABLE 5:** Regression analysis for policy support (*N* = 194).

Variables	*B*	β	*SE*	*p*	*R*^2^	Adjusted *R*^2^
**(Constant)**	**2.40**		**0.61**	**<0.001**	**0.24**	**0.20**
GPA regional	0.08	0.11	0.09	0.340		
PA regional traditional	0.03	0.04	0.06	0.611		
PA regional active	–0.06	–0.05	0.11	0.590		
**GPA global**	**0.28**	**0.29**	**0.07**	**<0.001**		
**Political orientation**	**−0.01**	**−0.18**	**0.00**	**0.008**		
**Environmental consciousness**	**0.01**	**0.24**	**0.00**	**<0.001**		
Duration	–0.01	–0.08	0.01	0.400		
Age	0.00	0.00	0.01	0.977		
Gender	–0.03	–0.01	0.16	0.869		

*GPA, general place attachment; PA, place attachment. Significant results displayed in bold.*

**TABLE 6:** Regression analysis for pro-environmental intentions (*N* = 194).

Variables	*B*	β	*SE*	*p*	*R*^2^	Adjusted *R*^2^
**(Constant)**	**4.72**		**0.53**	**<0.001**	**0.23**	**0.20**
GPA regional	–0.05	–0.07	0.08	0.511		
PA regional traditional	–0.06	–0.10	0.05	0.231		
PA regional active	0.05	0.05	0.09	0.568		
**GPA global**	**0.24**	**0.28**	**0.06**	**<0.001**		
**Political orientation**	**−0.01**	**−0.21**	**0.00**	**0.002**		
Environmental consciousness	0.01	0.10	0.00	0.165		
Duration	0.00	0.08	0.01	0.404		
Age	0.00	0.03	0.01	0.731		
Gender	0.01	0.01	0.14	0.934		

*GPA, general place attachment; PA, place attachment. Significant results displayed in bold.*

### Discussion

The third study employed an experimental repeated-measures design to test whether information derived from EO data affects feelings of place attachment, emotions, environmental policy support, and pro-environmental intentions. We tested whether seeing one’s place deteriorating as a result of climate change would alter the quality of place attachment people report. However, our main hypothesis that place attachment would decrease after being confronted with negative local EO information could not be confirmed. Place attachment remained stable between the two points of measurement, regardless of presented EO information, perhaps indicating that place attachment is a strong characteristic that cannot easily be changed using an image (rather than active imagination; see Scannell and Gifford, 2017; Reese et al., 2019). However, the presented satellite images differentially influenced people’s emotional experience: People who saw EO data presenting a change in landscape felt more worried, angry, guilty, and hopeless and experienced less joy and hope than people who saw no change. This effect was stronger for the global/control condition. This is interesting, as the change in the control region looked equally drastic but was in fact normal for that region, whereas the depicted change of the local environment was not normal. People who saw no change in their local area had little negative feelings and expressed more joy than people in other conditions. This partially contradicts our initial hypothesis, in which we expected stronger negative affective reactions when EO data depicted change of the home region compared to no change or change at another place. Finally, the intervention did not prove effective with regards to policy support or pro-environmental intentions; i.e., people who saw images of their local area changing in a negative but realistic way were less likely to support global policies for climate protection. This has potentially troubling implications: If making a problem (e.g., drought) visually perceivable is associated with less support for those policies that may prevent the problem from occurring, campaigning and communication strategies might reconsider some of their instruments. Further research is needed to investigate specifically why people may act in this counter-intuitive, perhaps defensive way. Independently of the presented EO data, global place attachment predicted both policy support and pro-environmental intentions. People with stronger negative emotions indicated more policy support.

## General Discussion

Global environmental change is coming – at large, and visibly in specific regions. This paper, presenting one experimental and two correlational studies, examines the role of place attachment and emotional responses in relation to regional and global place change, as depicted through EO data. At the intersection of EO and psychological science, this paper provides insight into how emotional bonds to place interact with visualized global and regional change. Without reiterating the depth of results, the set of studies suggests that stronger *global* place attachment in particular is related to stronger interest in EO data and consequences of climate change, while regional and national place attachment only correlated with interest on the respective levels. The second important finding is that global place attachment was most predictive of climate mitigation policy support, even beyond known predictors such as environmental consciousness or political orientation. Finally, we tested the effects of visualized environmental change on place attachment and emotional responses. While there was no effect on place attachment, there were effects on emotions such that people who were confronted with visualized environmental change showed stronger negative emotional responses.

### Place Attachment and How We Respond to Global Change

For several reasons, these findings are noteworthy. First, they show the necessity of bringing together environmental science (here EO science) and psychological responses to environmental changes that can be transmitted via visualization of EO data. We believe that this can bring forward downstream usage of EO data. Finding ways to make use of EO data on the level of citizens can improve policies derived from remote sensing data, as its effects on citizens can support policy makers in their decisions and communication strategies.

Second, our findings corroborate to research on the crucial role of global place attachment for pro-environmental action and climate mitigation (see also Devine-Wright et al., 2015; Walker et al., 2015). As in previous studies, it was an important predictor for pro-environmental policy support. Proximizing climate change in the form of EO data depicting change in the home region did not lead to increased emotional responses, policy support, or pro-environmental intentions. This is in line with other research suggesting that reducing psychological distance with climate change does not lead to favorable responses (e.g., Brügger et al., 2016). Taken together, this suggests that one motivating psychological feature is a shift of people’s minds from a local to a global consciousness vis-á-vis the global challenges humanity faces (Shwom et al., 2008). This shift can be based on an emotional bond to Earth as a whole, as is suggested in the current studies, but also as a bond to the group of all humans. Research on global identity for example suggests that the more strongly people identify with an in-group encompassing all humans, the stronger their pro-environmental behavior, attitudes, and pro-environmental policy support (e.g., Reysen and Katzarska-Miller, 2013; Rosenmann et al., 2016; Renger and Reese, 2017; Joanes, 2019; Loy and Reese, 2019). It is yet an open question whether it is the bond to Earth or to all humans that is more strongly tied to care and action for the planet. As a consequence, we believe that the current studies call for stronger theoretical integration of place attachment and social identity. The relationships we found in the current set of studies could possibly be explained – at least to a certain extent – with in-group identification. For example, it is likely that the finding that global rather than national place attachment predicts climate policy support would be the same for global vs. national identification. A recent theoretical model, the social identity model of pro-environmental action (SIMPEA; Fritsche et al., 2018), addresses under which conditions our social identities affect how we perceive and respond to environmental challenges. We believe it could serve as a potential framework for integrating place attachment and social identity research succinctly.

Third, we found for the first time that interest in EO data is linked to the bond to a place. Those people who identified strongly with their region were more interested in EO data of that place. Place dependence was independent of such interest. While place identity is linked to an emotional bond to one’s place and refers more to a symbolic meaning (Williams and Vaske, 2003), place dependence refers to the importance of a place in providing good conditions for activities or reaching one’s goals. Thus, the latter might be a more rational approach linked to physical characteristics of a place. The link between place identity and interest also supports the results of emotional responses to EO data showing changes in one’s place. In previous literature, too, place identity showed a stronger relation to pro-environmental behavior (Vaske and Kobrin, 2001) and policy support (Kyle et al., 2003) while place dependence did not show such a relation. Our study thereby shows that the research instrument used in place attachment research matters. Using different scales across the studies allowed us to observe that it is identification with a place rather than dependence. Yet, it would be useful to test whether the concept of place dependence required extension. Do people feel stronger place dependence on place and what it could offer in terms of eco-system services? If so, would this strengthen the relation between place dependence and interest in environmental change? These are questions for future research. And finally, interest in regional EO data is particularly high. People who feel a strong bond to their own region are more interested in EO data. This impact goes beyond global place attachment.

### Limitations and Future Research Directions

There are some limitations to the current research. Throughout our studies, we found evidence that place attachment and interest in EO data and planetary changes are related. However, we could not find unanimous evidence that such changes have a causal influence on place attachment.

We used a non-probable purposive sampling approach to recruit participants for all three studies leading to non-representative samples. Findings of these studies can thus not be generalized to other segments of the general population. Nonetheless, the samples in Studies 2 and 3 are relatively diverse and heterogeneous, compared with student samples that are often applied. We would also like to stress that addressing a specific region – as is often the case in place attachment research – makes it more difficult to generalize. However, we can show that in our specific region, of which many similar regions exist in terms of geography and affectedness, at least in Europe, place attachment seems to be an important predictor of interest in change and support for corresponding policies. Further, we did not control if participants had relations with the control region. This should be done in future studies to control for effects of place attachment to the control region. Further, future research should consider longer-term interactions of EO information and both local and global place attachment, policy support, and pro-environmental intentions. It is probable that our intervention was not strong enough to elicit detectable changes in place attachment – however, it cannot be excluded that the constant confrontation with EO information about local changes would in fact have more noticeable and relevant consequences. Thus, long-term, repeated-measures designs are warranted. It is further noteworthy that people in the Palatinate had experienced an unusually hot and dry summer, sparking public conversation about the local consequences of climate change for the first time. It is probable that the real experience of an entire summer was more meaningful than the presentation of images as part of a research study or people were already starting to get used to the extreme in their own region and thus paid more attention to changes in another place.

### Conclusion

We are facing a global environmental crisis that is becoming increasingly noticeable in previously unaffected regions and societies. This paper shows that belonging to a place matters as far as interest in changes of places are concerned. In how far a constant visualization of change can contribute to people’s behavior change in favor of climate protection remains an open question, however. This first set of studies provides a starting point for future research that should investigate how a changing planet changes our conceptions of place, and how we treat Earth. Creating a mind shift toward global place conscientiousness combined with local action opportunities might be a fruitful path toward societal change.

## Data Availability Statement

All datasets generated for this study are included in the article/Supplementary Material.

## Ethics Statement

Study 1 and Study 2 were conducted in line with the regulations of the German Psychological Association (DGPS) and the declaration of Helsinki. For Study 3, which included an experimental manipulation, was reviewed and approved by the Local Ethics Committee of Department 8 (Lokale Ethikkommission [LEK] des Fachbereich 8), Department of Psychology, University of Koblenz-Landau (application 153_2018). The patients/participants provided their written informed consent to participate in this study.

## Author Contributions

MW and LH: investigation, methodology, formal analysis, writing – original draft, and visualization. GR: conceptualization, funding acquisition, formal analysis, writing – original draft, and supervision. All authors contributed to the article and approved the submitted version.

## Conflict of Interest

The authors declare that the research was conducted in the absence of any commercial or financial relationships that could be construed as a potential conflict of interest.

## Footnotes

1 https://snapplanet.io/, last accessed 03/16 2020.

2 The analysis of these data is not part of the current research.
